# Natural and Synthetic Polymers for Biomedical and Environmental Applications

**DOI:** 10.3390/polym16081159

**Published:** 2024-04-20

**Authors:** Galina Satchanska, Slavena Davidova, Petar D. Petrov

**Affiliations:** 1 BioLaboratory, Department of Natural Sciences, New Bulgarian University, Montevideo Str. 21, 1618 Sofia, Bulgaria; stdavidova@nbu.bg; 2 Institute of Polymers, Bulgarian Academy of Sciences, Akad. G. Bonchev Str., Bl.103A, 1113 Sofia, Bulgaria; ppetrov@polymer.bas.bg

**Keywords:** polymers, natural, synthetic, biomedical, environmental

## Abstract

Natural and synthetic polymers are a versatile platform for developing biomaterials in the biomedical and environmental fields. Natural polymers are organic compounds that are found in nature. The most common natural polymers include polysaccharides, such as alginate, hyaluronic acid, and starch, proteins, e.g., collagen, silk, and fibrin, and bacterial polyesters. Natural polymers have already been applied in numerous sectors, such as carriers for drug delivery, tissue engineering, stem cell morphogenesis, wound healing, regenerative medicine, food packaging, etc. Various synthetic polymers, including poly(lactic acid), poly(acrylic acid), poly(vinyl alcohol), polyethylene glycol, etc., are biocompatible and biodegradable; therefore, they are studied and applied in controlled drug release systems, nano-carriers, tissue engineering, dispersion of bacterial biofilms, gene delivery systems, bio-ink in 3D-printing, textiles in medicine, agriculture, heavy metals removal, and food packaging. In the following review, recent advancements in polymer chemistry, which enable the imparting of specific biomedical functions of polymers, will be discussed in detail, including antiviral, anticancer, and antimicrobial activities. This work contains the authors’ experimental contributions to biomedical and environmental polymer applications. This review is a vast overview of natural and synthetic polymers used in biomedical and environmental fields, polymer synthesis, and isolation methods, critically assessessing their advantages, limitations, and prospects.

## 1. Introduction

Natural polymers extracted from organic sources such as microorganisms, algae, plants, or animals have been widely used for decades in biomedical applications such as pharmaceuticals, tissue regeneration scaffolds, drug delivery, and imaging [1]. Polysaccharides, proteins, and polyesters derived from plant and animal kingdoms are part of the family of natural polymers. Several of these polymers comprise our diet and have been used in various human applications [2]. These polymers are recognized by the biological environment and directed into metabolic degradation. Natural polymers are similar to extracellular matrix (ECM) components, enabling them to avoid chronic immunological reactions and toxicity, which are frequently observed with synthetic polymers [2].

**Figure 1:**
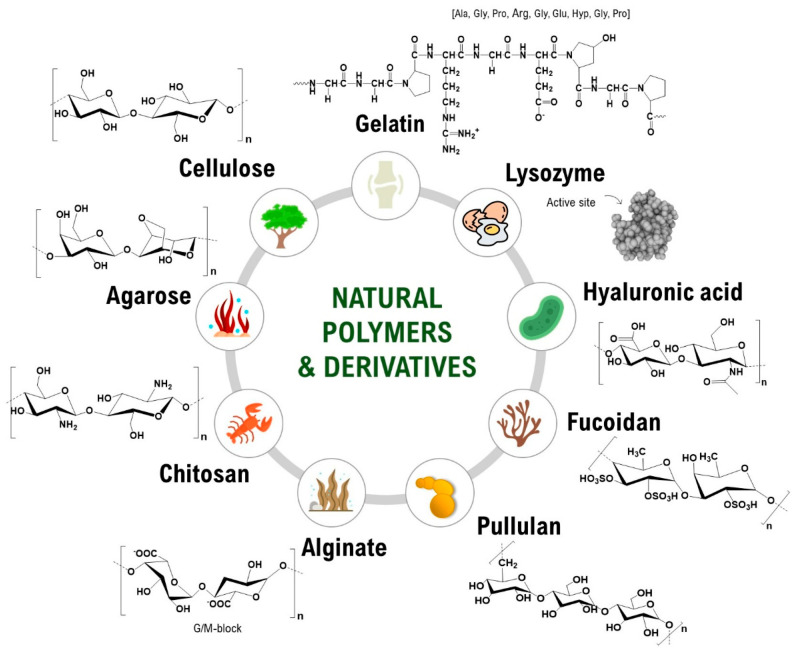
Examples of the natural polymers and derivatives used for materials [6].

Natural polymers are components of biological systems responsible for performing various essential functions [3]. For instance, specific natural polymers, such as cellulose and chitin, play a vital role in maintaining the structural integrity of cells in plants and animals. In contrast, others, such as lysozymes, offer biological protection against surrounding environments [4]. The diversity in their origin and composition provides these natural polymers with distinct physicochemical and biological properties and are of interest in various fields, e.g., in the manufacture of paper goods and textiles, as additives in food products, in the formulation of nutraceuticals and functional foods, and in the biomedical field (e.g., in cosmetic treatments and drug delivery) [5]. Their exploitation is favorable due to the natural abundance, renewability, and intrinsically low carbon footprint of polymers derived from renewable resources. Such properties are pivotal in developing advanced materials for films, membranes, coatings, hydrogels, and micro- and nanoparticle systems [6]. Natural polymers are essential for supporting life and enabling organisms to adjust to their surroundings through vital biological processes like molecular identification and genetic information transfer. Examples of natural polymers and their structures can be seen in Figure 1.

In recent years, natural polymers from marine resources have increasingly attracted attention, as they are more abundant and biologically active than polymers from other resources [7]. Marine sources, for instance, crustaceans, seaweeds, and algae, are rich in polysaccharides such as agar, chitin/chitosan, alginate, and glycosaminoglycans and thus exhibit exciting features and properties. For instance, chitin is a structural material in the exoskeletons of crustaceans and insects. Such marine-derived biopolymers constitute a platform for developing valuable advancements with environmental and economic advantages [7]. Marine polymers are becoming popular in the biomedical field due to their abundance and inherent features such as biocompatibility, biodegradability, and biological activity. However, some of these polysaccharides have limitations regarding solubility in water and organic solvents due to strong intra- and intermolecular hydrogen-bonded polymer chains [2]. As a result, this restricts their ability to be processed and converted into value-added matrices, including fibers, membranes, scaffolds, and nanomaterials. Therefore, searching for effective, eco-friendly, and feasible solvents is essential [2,7]. Polysaccharides are made up of sugars, called monosaccharides, that are linked together by O-glycosidic linkages. Some of their properties, such as solubility, viscosity, gelling potential, and surface and interfacial properties, are determined by differences in the composition of monosaccharides, types and patterns of linkages, shapes of chains, and molecular weight. Additionally, polysaccharides have various physiological functions, making them highly valuable for applications in tissue engineering and regenerative medicine [2].

Synthetic polymers are defined as polymers that are artificially produced in laboratories, also known as manufactured polymers [8]. They are classified as thermoplastic and thermoset polymers and elastomers. Some examples of synthetic polymers are polyethylene (PE), polystyrene (PS), polyamides (PA), poly (vinyl chloride) (PVC), polytetrafluoroethylene (PTFE), polyisoprene (PI), phenol formaldehyde resins, and many others. Polymers made from synthetic substances (monomers) derived from petroleum oil are often created in a controlled environment, and their backbone usually comprises carbon–carbon bonds [9]. Specific initiators and catalysts are used to initiate and accelerate the chemical reactions between monomers. Table 1 compares some of the properties and features of natural and synthetic polymers [9,10].

Synthetic polymers are omnipresent in society as textiles and packaging materials, in construction, and in medicine, among many other essential applications. Synthetic polymers are a highly versatile and diverse group of substances, many of which have been explicitly applied in drug delivery, for example, solubilizing agents, nanoparticulate formation, surface modification, drug carriers, diagnostic imaging agents, and implants [11]. In addition, some of these polymers show many biological activities in their own right (e.g., antitumor, antibiotic, antiviral, and antithrombotic activities, as well as inhibition of efflux pumps such as P-glycoprotein) [12].

**Figure 2:**
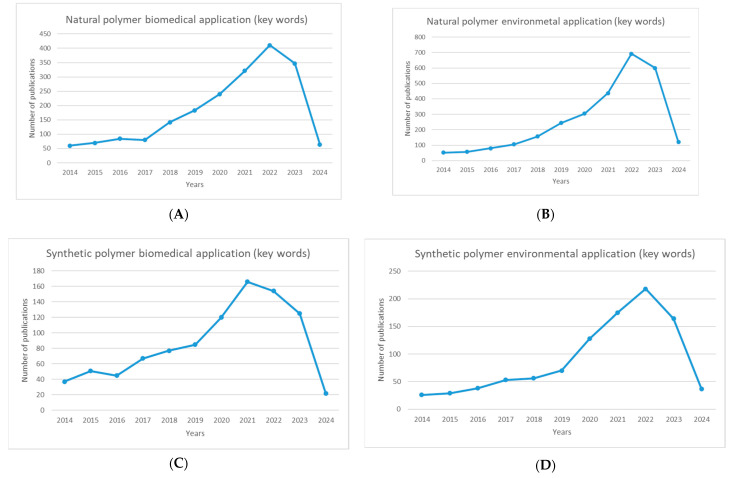
Scheme displaying four graphs showing the number of publications over ten years (2014–2024): (**A**) number of publications found using keywords “natural polymer biomedical application”; (**B**) number of publications found using keywords “natural polymer environmental application”; (**C**) number of publications found using keywords “synthetic polymer biomedical application”; (**D**) number of publications found using keywords “synthetic polymer environmental application”. Take into consideration that the graphs display only three months of 2024 (January, February, and March). The publications for ten years on consider the follwing: natural polymers for biomedical application (**A**) increased eight times; natural polymers for environmental application (**B**) increased 14 times; synthetic polymers for biomedical application (**C**) increased four times; synthetic polymers for environmental application (**D**) increased nine times. Based on the number of publications for 2024 so far (A-64, B-120, C-22, D-37), the tendency is expected to be maintained or even surpass the peak. The search was conducted using PubMed [13].

Natural and synthetic polymers have been widely discussed over the years, and interest in the topic has increased significantly in the last ten years. Figure 2 shows the increase in publications on natural and synthetic polymers and their use in biomedical and environmental applications.

Some of the main reviews for natural polymers for biomedical and environmental applications are [1,6,7,14,15,16,17,18,19,20,21,22,23,24,25,26,27,28,29,30,31,32,33,34,35,36] and for synthetic polymers [8,12,22,30,33,34,35,36,37,38,39,40,41,42,43,44,45].

## 2. Natural Polymers for Biomedical Use

### 2.1. Antibacterial

**Figure 3:**
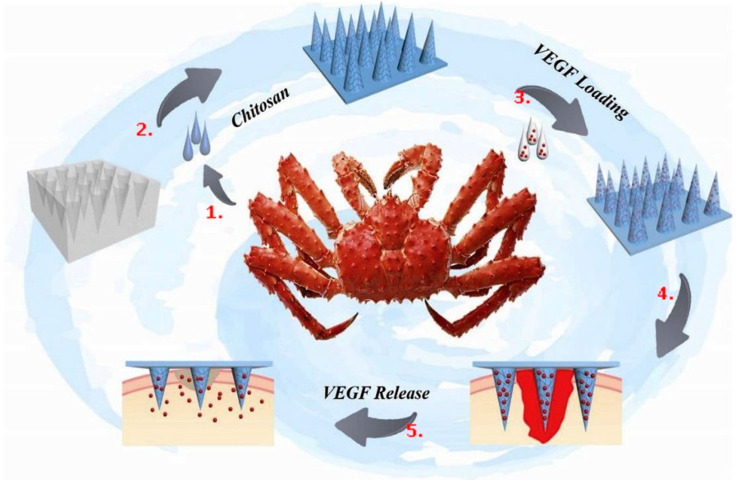
Scheme of the fabrication and controllable drug release application of the biomass microneedle patch [46]. No. 1: chitosan derived from the chitin of arthropods. No. 2: construction of chitosan microneedle array (CSMNA). No. 3: encapsulating active pharmaceutical molecules, vascular endothelial growth factor (VEGF), spotted in red, into the micropores of the CSMNA. No. 4: application of CSMNA onto an open wound. No. 5: controlled VEGF release from the CSMNA onto the skin, promoting wound healing.

Antimicrobial medication coatings, antimicrobial gauze or dressings, and medical tapes containing antimicrobial agents are a few examples of antimicrobial wound healing techniques. Chi et al. created a patch called the biomass-energetic chitosan microneedle array (CSMNA) to aid in healing wounds [46]. Due to its exceptional qualities and inherent antibacterial capabilities, chitosan is often utilized for wound healing [47]. The microneedle’s microstructure also helps to prevent excessive skin and patch adherence while delivering the drug-carrying agent to the target location. Meanwhile, a temperature-sensitive hydrogel wraps vascular endothelial growth factor (VEGF) in the CSMNA micropore [46]. As a consequence, the temperature rise brought on by the inflammatory response of the wound may be exploited to regulate the release of smart drugs. Biomass CSMNA patches have been proven in studies to support tissue regeneration, angiogenesis, collagen synthesis, and inflammatory control during wound healing [46]. Therefore, this multifunctional CSMNA patch may be helpful in clinical applications such as wound healing. A detailed scheme and explanation of the microneedle patch can be seen in Figure 3.

Zhang et al. suggested a novel class of controlled responsive particles for the release of drugs and the healing of wounds [48]. These hybrid particles comprised black phosphorus quantum dots (BPQDs) loaded with growth stimulants and antimicrobial peptides, gelatin, agarose, and filipin protein. The BPQDs absorb near-infrared (NIR) light and elevate the temperature of the particles to gelatin’s melting point when exposed to NIR radiation. The reversible phase transition (melting of gelatin) causes the enclosed medications to liberate gradually. BPQD-loaded particles with NIR-responsive characteristics have shown in vitro and in vivo investigations that they may accomplish the necessary regulated release of growth factors, hence encouraging neovascularization [48]. The particles were also antibacterial throughout storage and usage because the antimicrobial peptide was combined with a secondary hydrogel and enclosed in the scaffold. Due to these characteristics, BPQD-loaded natural protein hybrid particles are excellent for medication delivery and wound healing.

Silver nanoparticles (AgNPs) are often employed when making medical products like wound dressings. However, there is no agreement regarding the efficacy and safety of AgNPs. To establish the antibacterial impact of nanosilver in vivo and to assess the wound-healing capacity of silver-doped chitosan membranes, Shao et al. clarified the effects of proteins and inorganic ions on the antimicrobial characteristics of nanosilver [49]. Their antibacterial qualities and silver ion release patterns were assessed through in vitro interactions with a phosphate buffer or serum. In vivo tests were conducted to evaluate the antibacterial efficacy and wound-healing capacity of the systems. The findings demonstrated that the biological environment significantly impacts silver ions release: proteins are a barrier to prevent silver release, whereas inorganic ions cause delayed silver release. To achieve the in vivo antibacterial action, a high quantity of silver nanoparticles must be included. Additionally, embedding silver nanoparticles had no impact on the pace of tissue response or wound healing. It can be concluded that AgNP incorporation enhances the antimicrobial effect of biomaterials without modifying the wound-healing capacity of chitosan-based membranes [14].

### 2.2. Hydrogel Preparation and Application

Hydrogels prepared from natural polymers have attracted extensive attention in many biomedical fields, such as their use for drug delivery, wound healing, and regenerative medicine due to their excellent biocompatibility, degradability, and flexibility [15]. Hydrogels are three-dimensional networks formed by hydrophilic polymers through chemical cross-linking (covalent or ionic bonds) or physical cross-linking (hydrogen bonds, van der Waals forces, and physical entanglement) swollen in water [50,51].

**Figure 4:**
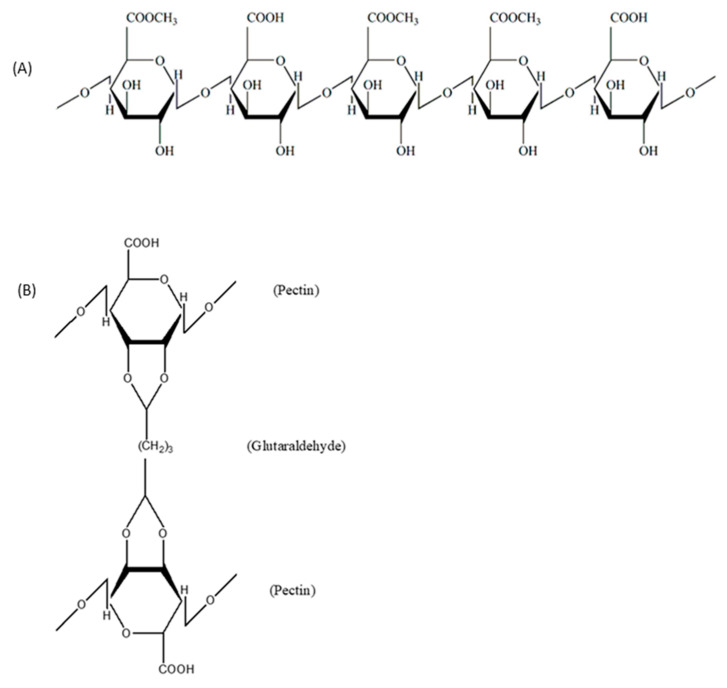
Chemical structure of (**A**) pectin and (**B**) pectin hydrogel film [52].

Hydrogels based on natural polymers such as alginate, starch, cellulose derivatives, chitosan, gelatin, collagen, hyaluronic acid, pectin, and so on show good degradability, biocompatibility, nontoxic degradation products, good flexibility similar to natural tissue, and have natural abundance, which endows them with widespread applications in medicinal fields, for instance, as drug carriers, wound dressing for wound healing, substrates for cell culture, cell delivery systems, scaffolds for tissue regeneration, and so on (Figure 4) [15,16].

Hydrogels based on natural polymers have emerged as promising alternatives for the ECM in biomedical applications due to their unique integration of biodegradability, biocompatibility, mechanical property tunability, biomimicry, and responsiveness, which could provide microenvironments with the preservation of cellular functions, promotion of cell health, and encouragement of tissue formation [53].

### 2.3. Drug Delivery

Extensive research has been conducted on the use of natural polymers as carriers for drugs and other bioactive substances, which has garnered a great deal of interest. Natural polymers have inherent advantages such as marvelous biocompatibility, controlled enzyme degradation, specific interactions with some biomolecules [54], and easy modification, which make them versatile for drug delivery applications. In this context, drug delivery systems (DDSs) constructed using representative natural polymers such as polysaccharides (chitosan and hyaluronic acid) and proteins (silk fibroin and collagen) are generally summarized. These DDS systems are used to deliver payloads, which mainly include low molecular weight drugs, proteins, and DNA, and are employed for various applications such as tissue engineering, wound healing, or anticancer therapy [55,56]. Moreover, a DDS constructed by modified biopolymers has also been presented, focusing on the chemical and morphological modifications, the additions of smart stimuli-triggered or targeted motifs, and so on, which promoted delivery and therapy efficiency.

Polysaccharides and protein-based materials show more similarities to the extracellular matrix, thus endowing natural polymer-based drug carriers with minimally invasive properties [17]. Moreover, polymer chains are abundant in some groups with accessibility to modification, including amino groups, carboxyl groups, hydroxyl groups, and so on, enabling easy accessibility for further modifications [17]. Finally, with more profound research into life science, more and more specific interaction behaviors between native polymers and organs or cells have been pointed out. Some natural polymers have shown a higher affinity to the receptors of cells and regulate cellular processes, including adhesion, proliferation, and migration, which provides promising potential for designing more specific target usages of high efficiency [57]. Moreover, their degradation behavior in the presence of enzymes in vivo also ensures their ability to construct stimuli-responsive systems for the delivery of drugs in the local sites. This perspective sheds light on the following two kinds of polysaccharides (chitosan and hyaluronic acid) and the other two types of proteins (silk fibroin and collagen) involving the delivery systems constructed by the original polymers and their derivatives [57].

Polymer/metal-organic frameworks (MOFs) are a class of crystalline materials possessing structures formed from the coordination of metal ions to multidentate organic groups. The main characteristics of MOFs are the high degree of porosity and the tunable architecture of the structure obtained by selecting appropriate metal ions and linkers. Furthermore, the surface of MOFs can be modified additionally, thereby increasing their functionality [58]. The high surface areas and large pore sizes of MOFs favor the encapsulation of high drug loadings. In contrast, MOFs’ high structural and functional flexibility allow their adaption to the drug molecules’ shape, size, and functionality. When a MOF is combined with a polymer, its colloidal stability is enhanced without loss of crystallinity. However, a recurrent issue is the decrease of porosity due to the polymer obstructing the entrance to the pores or the penetration of the polymer chains inside the MOF cavities [58]. In addition to increasing the stability, the polymer coating offers the possibility of adding targeting functionalities or introducing a stimuli-responsive release, allowing for the preparation of improved drug delivery or imaging devices. Some of the natural polymers used are hyaluronic acid, gelatin, chitosan, and alginate. Hyaluronic acid, for example, has been used to increase the binding affinity of nanoparticles selectively for the surface of cancer cells and was found to mediate the targeting recognition of CD44 over-expressing cancer cells [58]. Hyaluronic acid has incomparable chemical–physical properties, and numerous biological functions characterize it [18,19]. Also, hyaluronic acid has excellent antioxidants, good viscoelastic properties, excellent gelling properties, anti-inflammatory properties, wound-healing activity, excellent cosmetic properties, and drug carrier ability. Therefore, it is widely used in pharmaceutical, cosmetic, and biomaterials production industries. Hyaluronic acid has also recently been explored as a drug-delivery agent via different methods, such as nasal, oral, pulmonary, ophthalmic, topical, and parenteral [18,19].

### 2.4. Stem Cell Morphogenesis

Polymeric materials have great potential in tissue engineering thanks to their biodegradability, processing, and property design flexibility [59]. Moreover, polymers may be used to regulate cell function. Stem cells are a promising option for tissue engineering since they uniquely self-renew and differentiate into various lineages, such as neurogenic, osteogenic, chondrogenic, and myogenic, under proper stimulation from extracellular components [59]. Due to their properties, stem cells and polymeric materials are critical design choices. Stem cells can self-renew and commit to specific cell lineages under appropriate stimuli. Polymeric materials are biocompatible, degradable, and flexible in processing and property design. Therefore, a significant focus of tissue engineering is to utilize polymers, or soft materials, to control stem cell function via physical, chemical, mechanical, and biological cues “communicated” from the polymer to the cells [60]. Examples of natural polymers include collagen, fibrin, and polysaccharides, such as hyaluronic acid and alginate [60]. Polymers found in nature consist of diverse biological cues that include sequences for cell adhesion. Consequently, they are capable of being identified by cells. However, natural polymers are subject to batch-to-batch variation due to their structure and chemical composition complexity, leading to variations in tissue engineering outcomes [60].

There are at least two advantages of using biopolymeric materials for tissue regeneration. First, the structure and composition of polymers can be easily tailored to give rise to various physical and chemical properties that can promote certain cellular functions, including proliferation and differentiation, in a controlled manner [60]. Second, many polymers are biodegradable through either hydrolysis or enzymes secreted by cells. Therefore, over a prescribed time, the scaffold can be replaced by newly formed tissue. Thus, with degrading polymers, secondary surgery is unnecessary to remove the scaffold after implantation [60]. Polymeric materials are usually encapsulated by a layer of fibroblasts, collagen, and inflammatory cells in vivo, which is suboptimal for tissue formation. However, the biocompatibility of polymer materials can be improved by engineering the functionality of these materials. The behavior of stem cells can be controlled by engineering functionality into a biomaterial, such as via immobilization of adhesion peptides, modification of surface chemistry, and mineralizing polymer surfaces [60].

### 2.5. Wound Healing

Natural polymers play significant roles in different skin wound healing processes, contributing to the overall effectiveness of wound management and tissue repair. Natural polymers such as chitosan and hyaluronic acid can help reduce inflammation in the early inflammation phase of wound healing. With its anti-inflammatory properties, chitosan can minimize the inflammatory response, while hyaluronic acid contributes to a balanced immune response, potentially reducing excessive inflammation [14].

Natural polymers like collagen, chitosan, and keratin provide a scaffold for cell migration and proliferation. Collagen-based dressings act as a structural framework for cells to move into the wound area and stimulate cell division, promoting granulation tissue formation [14]. As a primary component of the extracellular matrix, collagen facilitates the formation of this supportive network. It guides fibroblasts to synthesize new collagen, helping reestablish tissue integrity. Hyaluronic acid and alginate maintain a moist wound environment conducive to cell proliferation and migration. This wet environment also helps prevent the formation of scabs, promoting faster healing. Collagen and gelatin contribute to collagen deposition and organization during the remodeling phase [14,46]. Collagen-based dressings and scaffolds help ensure the proper alignment and bundling of collagen fibers, improving the tensile strength of the healing tissue. Specific natural polymers, such as keratin, have been found to minimize scarring and promote a more natural appearance of healed tissue [14,15]. This is particularly valuable in aesthetic areas or where scar formation could impair function. Chitosan has inherent antimicrobial properties, helping prevent infections in the wound area. Chitosan dressings can inhibit the growth of bacteria, making them suitable for wounds at risk of infection. Alginate dressings, composed of seaweed-derived alginic acid, absorb excess exudate from the wound. This maintains a moist environment and helps prevent bacterial proliferation in overly damp conditions [61].

**Figure 5:**
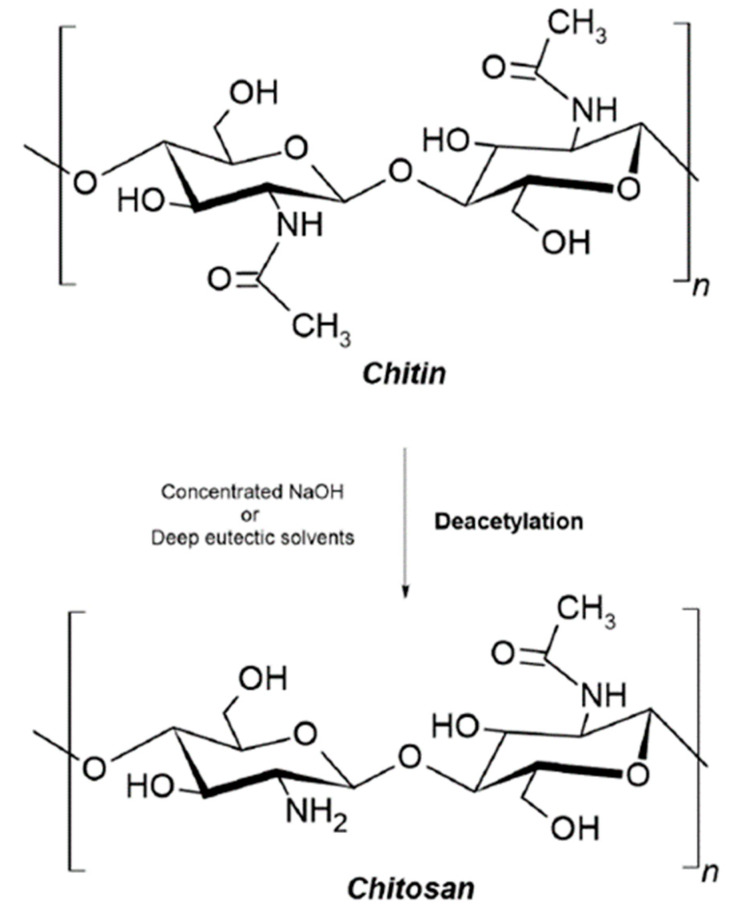
Deacetylation of chitin to chitosan [20].

Some natural polymers can enhance their bioavailability and activity when used as carriers for growth factors. This can further stimulate cell proliferation and tissue regeneration. For instance, hyaluronic acid can be a carrier for growth factors like epidermal growth factor (EGF). Natural polymers can stimulate angiogenesis (forming new blood vessels) by influencing growth factors and cell behavior. This is vital for ensuring adequate blood supply to the healing tissue. Polymers like pectin and chitosan, which can be used in wound dressings, create a protective barrier over the wound, allowing for oxygen and nutrient exchange (Figure 3 and Figure 5) [61].

This can help prevent external contaminants from entering the wound. Natural polymers play multifaceted roles in different phases of skin wound healing. They support and optimize the processes of inflammation, proliferation, and tissue remodeling, promote a favorable wound environment, reduce inflammation, prevent infections, and enhance tissue regeneration. Their biocompatibility and biodegradability make them valuable components of wound care strategies, with applications in various types of skin wounds, ranging from acute injuries to chronic ulcers and surgical incisions [14,61].

### 2.6. Skin Tissue Engineering

Due to their excellent biocompatibility, biodegradability, and low cytotoxicity, as compared to synthetic polymers, natural polymers find extensive application in skin tissue engineering [62]. Polysaccharides and protein-based materials are the two primary categories of natural polymers employed in hydrogels for this purpose. Dermal substitutes comprising collagen or hyaluronic acid serve as scaffolds for cellular growth. On the other hand, epidermal substitutes, consisting of keratinocytes and fibroblasts, replace the outermost layer of the skin [21].

### 2.7. Bone Tissue Engineering

Natural polymers, including alginates, collagens, hyaluronic acid, and gelatin, are commonly used in bone tissue engineering. These polymers are employed in three primary forms: nanofibrous scaffolds, hydrogels, and microspheres. Biocomposites have also been developed for bone tissue engineering by combining natural polymers with hydroxyapatite. Bone scaffolds serve as a crucial application of natural polymers and provide a supportive structure for cellular growth [21]. Osteogenic differentiation, involving transforming mesenchymal stem cells into bone-forming osteoblasts, is another essential aspect of natural polymer utilization. Additionally, natural polymers are used in bone regeneration strategies, acting as scaffolds or carriers for growth factors to promote the restoration of damaged or lost bone tissue [21,22].

### 2.8. Cartilage Tissue Engineering

Cartilage is composed of thick proteoglycans and collagen. This thick and lubricated structure presents particular challenges for adhesives and bonding strategies. Furthermore, cartilage defects lack a regenerative capacity, as they lack blood vessels/neural tubes. Natural polymers, such as collagen, chitosan, gelatin alginate, silk fibroin, and hyaluronan, have extensive applications in cartilage tissue engineering [21]. Cartilage scaffolds serve as primary natural polymers in cartilage tissue engineering, providing a supportive structure for cellular growth. Various materials can fabricate these scaffolds, including chitosan, collagen, alginate, silk fibroin, hyaluronan, and gelatin. Chondrogenic differentiation is another significant application of natural polymers involving transforming mesenchymal stem cells into chondrocytes, contributing to cartilage formation. Furthermore, natural polymers are being investigated for repair and regeneration techniques to promote the restoration of damaged or lost cartilage tissue. These techniques often employ natural polymers as scaffolds or carriers for growth factors [21].

### 2.9. Heart Valve Tissue Engineering

Polysaccharides are the most abundant biomaterials in nature and meet several criteria for eligibility for tissue engineering, which include biocompatibility, biodegradation, and the ability to support cell development. Due to their biological properties and structural and functional similarities to ECM, it is reasonable to use them in tissue engineering [23]. Polysaccharides become essential to promote heart valve tissue regeneration in combination with appropriate cells or bioactive molecules. Their applications for heart valve tissue engineering are vast and varied, and approximately 70% of all studies in this field focus on chitosan, alginate, hyaluronic acid, and cellulose, respectively [23].

### 2.10. Cell Encapsulation

**Figure 6:**
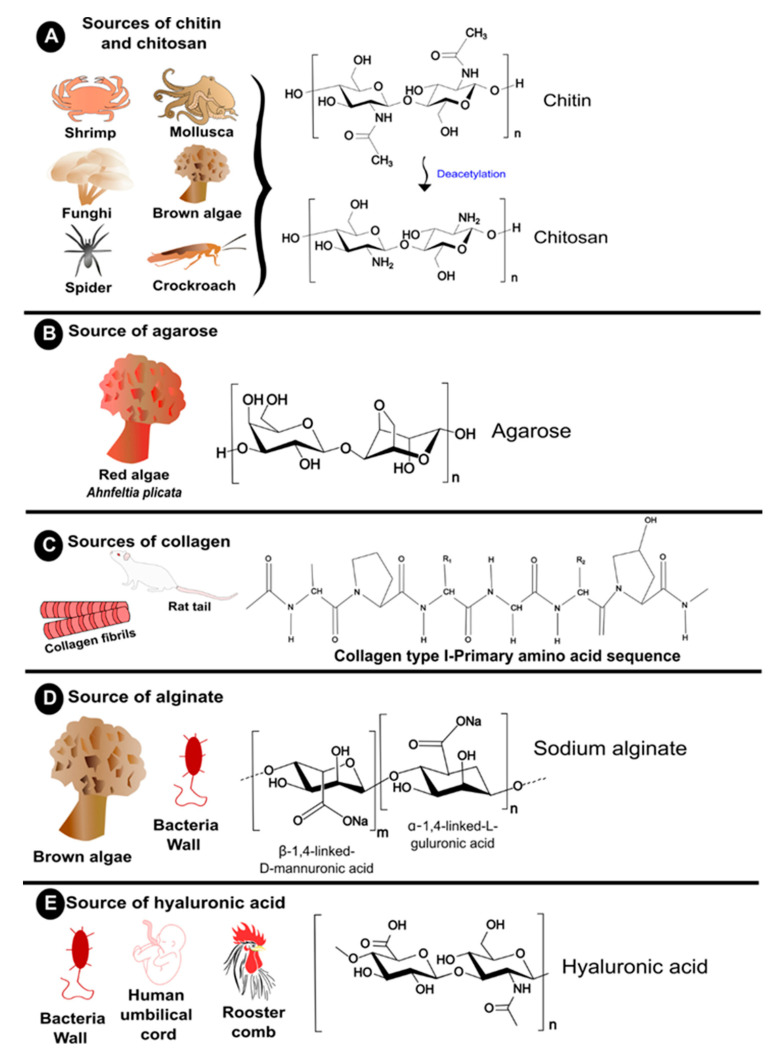
Schematic figure concerning the primary source of the most frequently employed biopolymers for cell microencapsulation and their polymer structures. (**A**) Chitin and chitosan. (**B**) Agarose. (**C**) Collagen. (**D**) Alginate. (**E**) Hyaluronic acid [24].

Cell encapsulation instead of therapeutic product encapsulation leads to longer delivery times, as cells continuously release encapsulated products. Moreover, cell encapsulation allows for the transplantation of non-human cells, which may be considered an alternative to the limited supply of donor tissues. In addition, genetically modified cells could also be immobilized to express any desired protein in vivo without host genome modifications [63]. Cell immobilization displays a significant advantage compared to protein encapsulation, allowing for the sustained and controlled delivery of de novo-produced therapeutic products at constant rates, leading to physiological concentrations. The versatility of this approach has adapted its use in treating diabetes, cancer, central nervous system diseases, heart diseases, and endocrinological disorders, among others. Hydrogels are among the most promising biomaterials for recreating native extracellular matrix (ECM) properties due to their high water content, biological compatibility, and moldability (Figure 6) [24].

Alginate is the most studied material for cell encapsulation and has been adopted for many biomedical applications. Alginate has historically been used as a protective barrier to enhance cell therapies for immunoprotection of pancreatic islets, treatment of brain tumors, treatment of anemia, and cryopreservation [64]. Current treatments include surgery, immunotherapy, chemotherapy, targeted therapy, hormone therapy, and radiation therapy and result in numerous adverse effects affecting the patient’s health, making the search for alternative therapies an emerging need [24,64]. Another critical study reported that liposomes, vesicles formed by phospholipids encapsulated in an alginate matrix, were transported directly and could release drugs directly to the colon cancer target, reaching higher drug concentrations in the tumor. Thus, in addition to comprising an alternative to cancer treatment, one of the most common diseases worldwide with high mortality rates, the alginate biopolymer may also be used as a carrier agent for targeted drugs [24].

### 2.11. Biofabrication

Recent advancements in biofabrication techniques allow the production of a polymer matrix biophysically and structurally similar to the ECM. Combined with different cell lines, this matrix can proliferate and differentiate into the desired tissue. Moreover, incorporating different growth factors or other biomolecules can improve the cells’ migration, growth, and differentiation [1,15].

Numerous research studies for polymer matrix biofabrication follow two different strategies for cell incorporation: (i) cell implantation on a previously formed polymer matrix and (ii) fabrication of a polymer matrix with encapsulated cells.

The first strategy has been used for the last decade and is restricted to cell implantation. Typically, these techniques do not enable effective assimilation between cells and the polymer matrix. The success of these methods in regenerating tissues depends on the polymer matrix’s physical characteristics, such as its degradation rate, hydrophobicity, and stiffness. The most used techniques are layer-by-layer melt molding, photolithography, and self-assembly [1].

The second strategy has been implemented in recent years as it allows the fabrication of advanced cell-laden structures with complex cellular microenvironments. Recently, advanced techniques, such as microfluidics, electrospinning, and 3D bioprinting, have permitted the integration of cells directly into the polymer matrix with accurate physical and biological properties to match the ECM of the desired tissue [1].

### 2.12. Bio-Based Monomers

Itaconic acid, a promising bio-based monomer material, can be obtained using fermentation. Baup discovered it in 1836 while conducting the pyrolysis of citric acid. However, it was only in 1932 that it was reported as a biological product synthesized by *Aspergillus itaconicus* [18]. Due to its non-toxicity, biocompatibility, biodegradability, chemical reactivity, and microbe resistance, it has excellent potential for various scientific uses in biomedical, food, agricultural, pharmaceutical, and other industries [18,25].

Georgius Agricola discovered succinic acid in 1546 [18]. Succinic acid is a C-4 dicarboxylic acid, considered one of the most promising bio-based monomers for producing microbial fermentation. It has been used in the food industry and is derived from various microorganisms and agricultural carbohydrates; it is non-toxic, biocompatible, and biodegradable. Thus, it is widely used in developing biomedical products, food additives, pharmaceutical products, surfactants, detergents, microbe-resistant products, green solvents, and biodegradable plastics [18,65].

Citric acid production on an industrial scale began in 1890, thanks to the Italian citrus fruit industry. In 1917, James Currie, an American food chemist, discovered a way to produce citric acid using *Aspergillus niger* [18]. Two years later, Pfizer, a pharmaceutical company, started using this technique for industrial production [18]. Citric acid is a natural organic compound involved in the Krebs cycle. It is multifunctional, nontoxic, biocompatible, and biodegradable. It finds widespread use in the chemical, food, cleaning, and biomaterials production industries [18,66]. While the industrial applications of citric acid are well known, the biomedical applications of chemically and physically modified citric acid or cross-linked polymer biomaterials have not been thoroughly reviewed.

Microbial fermentation produces glutamic acid, a biodegradable natural bio-based amino acid monomer. In 1866, Karl Heinrich Ritthausen, a German chemist, discovered and identified glutamic acid by treating wheat gluten (the substance’s namesake) with sulfuric acid [18]. Glutamic acid plays a crucial role in the body’s disposal of excess or waste nitrogen and undergoes oxidative deamination catalyzed by glutamate dehydrogenase. Because of its non-toxicity, biodegradability, biocompatibility, and excellent cation chelating ability, glutamic acid has various applications in various industries, such as pharmaceuticals, cosmetics, food, water treatment, and agriculture [18,67]. Poly (glutamic acid) (PGA) is a natural linear polymer synthesized by bacilli like *Bacillus subtilis*, formed by the peptide bonds between the α-amino group and the γ-carboxyl group at the end of the glutamic acid side chain. Biomaterial development has extensively utilized glutamic acid due to its excellent bioactive properties, which can be achieved by chemical and physical modification or cross-linking with natural and synthetic polymers [18,67]. Table 2 summarizes the characteristics of the natural bio-based monomers mentioned.

## 3. Natural Polymers for Environmental Use

### 3.1. Food Packaging

Various natural polymers from renewable sources have been used to develop biobased food packaging. The primary natural sources used for packaging are derived from polysaccharides, lipids, proteins, or blends of these polymers. The utilization of these natural materials is linked to their biodegradability and renewability [26]. However, other advantages are expected when used for food packaging. For example, these materials can act as carriers of functional substances, add well-being benefits, incorporate flavorings and colorings, enhance organoleptic characteristics, improve mechanical and barrier resistance, etc.

Polysaccharides possess suitable oxygen barriers and have sites for hydrogen bonding formation, which can be used to incorporate functional substances, e.g., coloring, flavoring, and antioxidant agents. In contrast, these materials do not exhibit an excellent barrier to water vapor, which can be overcome by blending with other hydrophobic substances, such as lipids. Polysaccharides have been used to develop natural-based packaging [26,27].

Plant-derived proteins have gained the remarkable attention of food manufacturers and consumers searching for natural food resources and alternative materials to vegetarian, vegan, and food allergy diet restrictions. Protein-based film packaging exhibits extraordinary mechanical and barrier properties, especially against oxygen and carbon dioxide gases, compared to polysaccharides. In addition to being eco-friendly materials, these films can nutritionally improve food quality and preservation. Moreover, the amphiphilic attribute of proteins contributes to their utilization as emulsifiers by stabilizing the oil/water interface due to changes in interfacial tension. Many plant proteins, such as soybean, wheat, corn, sunflower, and peas, are used in the food industry and packaging [27].

Edible films can be defined as a thin layer of a material coating or placed between foods, which act as a barrier and that can be consumed without any health risk. Both are primary food packaging and can be considered similar, although they differ substantially. Mainly, films are solid laminates, separately prepared, dried, processed, and then used to cover food surfaces, placed between food parts, or used as an edible sealed bag. Differently, coatings are prepared as a solution, directly sprayed or dipped on the food surface, and then dried. Thus, coatings can be considered part of the food product since they are not made to be removed. Moreover, films can be prepared as mono, bi, or multilayers. The latter provide a better water barrier to food but are less commonly used as they need two or more casting and drying processing steps [20].

Both films and coatings should be composed only of food-grade components, GRAS (generally recognized as safe), including any additives, such as plasticizers. As with other packaging, these materials must protect the food’s integrity and quality. Although edible films are not expected to replace all conventional packaging, they can significantly reduce the use of petroleum-based plastics, decrease food losses, and reduce environmental pollution over long periods [28].

### 3.2. Nano Fertilizers and Micronutrients

**Figure 7:**
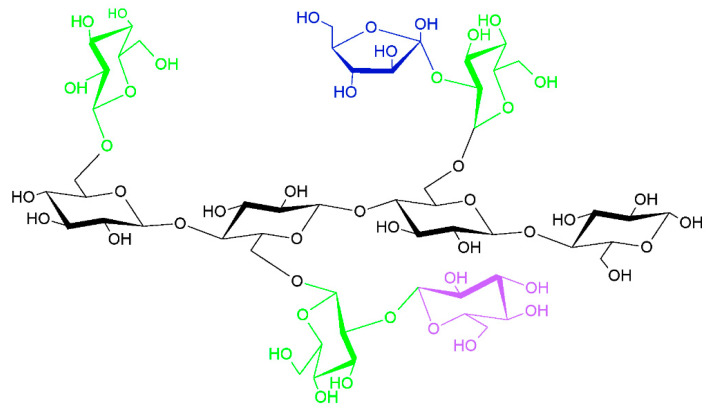
Representation of molecular structure of hemicellulose (glucose units in black, xylose in green, galactose in violet, and arabinose in blue) [68].

Biopolymers such as alginate, cellulose, chitin, chitosan, hemicellulose, lignin, polypeptides, and polyesters, used as nanocarriers to encapsulate nutrients and avoid dissolution and oxidation, are an eco-friendly option due to their natural origins and are biodegradable when compared to bulk synthetic fertilizers (Figure 7).

Chitosan is the most accepted biopolymer for agriculture due to its innocuous origin and ability to protect plant cells. It is also an easy-to-manipulate matrix to program the adsorption and slow release of the target active ingredient. The cover of nano fertilizers is designed to be porous for the slow release of the nutrient content. The time and dose of nutrient release will depend on the plant’s requirement [29,69].

Commercial nano nutrients offer advantages such as controlled release due to the cover materials of the mentioned fertilizers. Controlled release refers to the slow delivery of the nutrient over months. Some available products coated with patented biopolymers are Agrocote (ICL Group Ltd., St. Louis, MO, USA), ESN Smart nitrogen (Nutrien Ltd., Joplin, MO, USA and Saskatoon, SK, Canada), Meister (OCP Ltd., Clayton, Australia), Multicote (Haifa Group Ltd., Israel, with offices in five continents), Nutricote of Florikan CRF (Florikan Ltd., Sarasota, FL, USA), and Osmocote (ICL Group Ltd.); zeolites are generally used as fertilizers for fruit trees, coffee beans, bananas, sugar cane, vegetables, potatoes, rice, corn, and wheat, among others [29].

**Figure 8:**
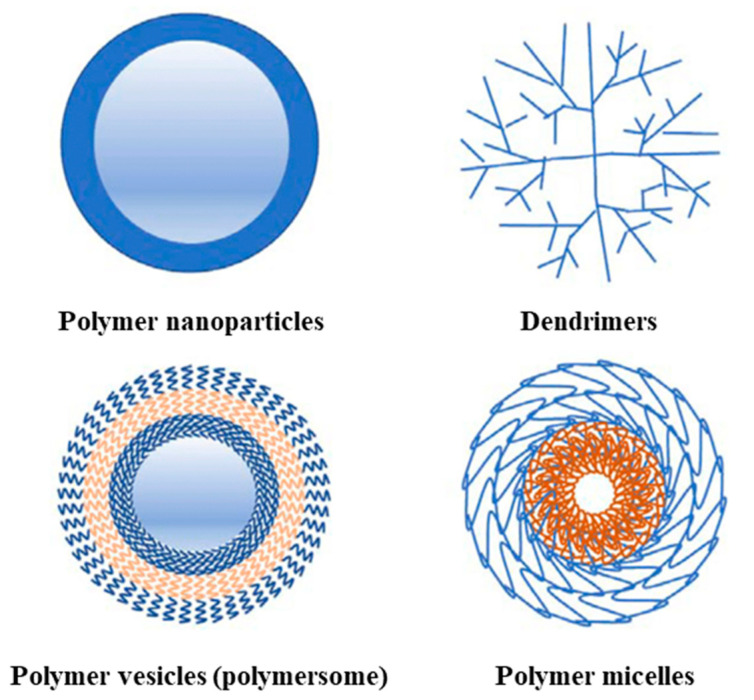
Different types of polymer-based nanocarriers [30]. Upper left: Polymer nanocapsule is a microcapsule in which drugs are present in a core (light blue color) surrounded by a polymer shell (dark blue color); Upper right: Dendrimers are highly branched polymer molecules consisting of a core and branches connected around the core <100 nm in size; Lower left: Polymer vesicle membranes are composed of special amphiphilic block copolymers, biomimetic analogs of natural phospholipids, ranging in size from 10 nm to 10 µm. The hydrophobic segments (yellow color) of copolymers gather together to reduce contact with water, while the hydrophilic groups are distributed on the outer side of the membrane (dark blue color), thus forming a typical bilayer membrane structure similar to that of liposomes; Lower right: In polymer micelles, hydrophobic drugs are encapsulated in the hydrophobic core (yellow/orange color), while the hydrophilic shell (blue color) plays a role in maintaining particle stability, which makes it suitable for intravenous injection.

Finally, biopolymers as nanocarriers of nano fertilizers or micronutrients have advantages over conventional fertilizers, such as the low amount applied and the controlled nutrient release, which is more profitable for increasing crop production and fruit quality. Therefore, the future of nano fertilizers is promising because of the ecological approach [29]. A scheme of the different types of polymer-based nanocarriers is presented in Figure 8.

### 3.3. Nanocarriers of Fungicides/Bactericides/Viricides

Although other polymers can be applied in agriculture, chitosan is one of the essential enhancers of plant defenses, as this biodegradable polysaccharide hydrogel forms protection barriers in plants and helps the plant develop defense responses against pathogens. These properties of chitosan-based obstacles have been tested with excellent efficacy against fungi and oomycetes [29]. Moreover, chitosan can be combined with other materials (e.g., montmorillonite) to encapsulate nutrients or active ingredients of pesticides. Additionally, hydrogels containing plant repellents (essential oils) encapsulated in nanoparticles were fabricated for plant protection. Some reports showed chitosan-based micelles as a controlled-release formulation for biosafe pesticide delivery [29].

### 3.4. Nanocarriers of Insecticides

Polymer-based materials have also been found to be effective insecticide carriers, mainly by increasing their solubility in water. Microspheres composed of chitosan and cashew tree gum were developed and loaded with the essential oil of *Lippia sidoides*, active against larvae of *Aedes aegypti*, to use as a bioinsecticide to control larvae proliferation. These chitosan-based capsules showed a prolonged larvicidal effect. Similarly, microcapsules of alginate and chitosan were found to be suitable matrices to carry nano imidacloprid bioinsecticide. Interestingly, this carrier system allowed for up to eight times longer insecticide release when compared with an insecticide used alone [29].

Moreover, release time depends on the concentration of alginate and chitosan used for encapsulation. The amphiphilic derivative of chitosan, *N*-(octadecanol-1-glycidyl ether)-O-sulfate chitosan, was used to form spherical polymeric micelles (167–204 nm size) for the encapsulation of insecticide. These nanoparticles were formed by self-assembly in an aqueous solution, increasing the 1300-fold solubility of rotenone in water and providing sustained release. The development of carboxymethyl chitosan nanoparticles with ricinoleic acid as an emulsifier for azadirachtin was helpful as an insecticide agent for agricultural applications due to the slow release of the active compound. These spherical particles in a size range of 200–500 nm showed good polydispersion, smooth high zeta potential, and solubilization in the water of the lipid-soluble azadirachtin [29].

### 3.5. Bioplastics

**Figure 9:**
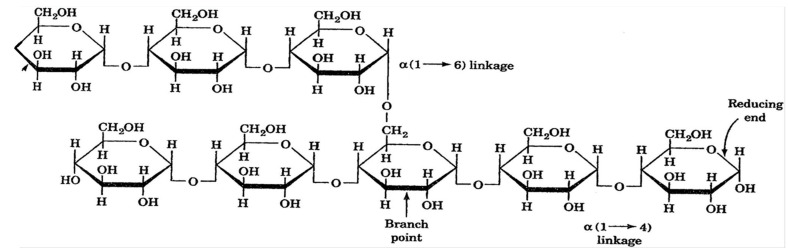
Structure of starch [71].

A material can be considered a bioplastic if it possesses the following properties, as per the European Bioplastics Organization (EBO): it is either bio-based, biodegradable, or has both characteristics. Bio-based refers to materials or products derived wholly or partially from renewable resources (biomass), thereby replacing the petrochemical resin typical of conventional plastics with vegetable or animal polymers. Natural fibers, such as wood fibers, hemp, flax, sisal, and jute, replace compounds like glass carbon fiber or talc. Agro-polymers-based bioplastics are made from well-known feedstocks such as starch, cellulose, pectin, and animal and vegetable proteins, such as casein and gluten (Figure 9) [70].

Polysaccharides such as cellulose and pectin can be obtained from various fruits and vegetables such as potatoes, corn, rice, tapioca, and apples. These are primarily used in the manufacture of packaging materials [72]. Protein additives are frequently utilized to create materials with new or enhanced technological features. The elemental compositions of proteins (covalent bonds between hundreds of amino acids) and polysaccharides (covalent bonds between ramifications of monosaccharides) differ. Thus, when mixed, they can exhibit different physicochemical and rheological properties, resulting in a wide range of two- and three-dimensional structures.

In addition, many polymers could be produced by various microorganisms cultured under different nutrient and environmental conditions. Polyhydroxyalkanoates (PHAs) are linear thermoplastic polymers with hydroxyalkanoic acid as a monomer unit [31]. They can be synthesized intracellularly as insoluble cytoplasmic inclusions by heterotrophic bacteria, such as *Cupriavidus necator*, recombinant *Escherichia coli*, and photoautotrophic microorganisms like microalgae. Their synthesis occurs due to excess carbon when other essential nutrients, such as oxygen, nitrogen, or phosphorus, are restricted. After their extraction from cell cultures, they can be processed similarly to polypropylene, including extrusion and injection molding, obtaining a material with similar properties [31].

Bacteria can also be used to produce biodegradable polymers through the fermentation of carbohydrates obtained from agricultural by-products such as sugar, corn, wheat, and corn starch. Poly(lactic acid) (PLA)-based bioplastics are obtained from a fermentative process that involves converting carbohydrate sources into dextrose, followed by fermentation or conversion into lactic acid. Therefore, lactic acid is isolated and polymerized to yield a low molecular weight, brittle polymer whose chain length could be increased using external coupling agents [31].

## 4. Methods of Isolation and Physicochemical Parameters of Natural Polymers

In earlier days, biopolymers were isolated from agricultural feedstock such as corn, potatoes, and other food residues by chemical processes. Still, due to biotechnological developments, the focus has shifted to renewable sources other than food, like cellulosic biomass, by various enzymatic and bacterial fermentations. In addition to these plant biopolymers, bio-based polymers from animal sources such as proteins, nucleic acids, collagen, and chitosan have shown a mammoth increase due to recent technological and commercial process improvements [73].

Several methods are available to isolate biopolymers from these natural sources. Two basic principles are followed to make biopolymers from raw sources: acid or alkaline hydrolysis, the production of monomers by chemical modification/bacterial fermentation, and enzymatic processes. For the efficient production of biopolymers, it is necessary to characterize them using different methods. There are different physicochemical characterization methods available for each biopolymer to identify the molecular properties, morphology, isoelectric points, and functional group by methods such as scanning electron microscope (SEM), X-ray diffraction (XRD), gel electrophoresis, Fourier transform infrared (FTIR) spectroscopy, etc. [73].

**Figure 10:**
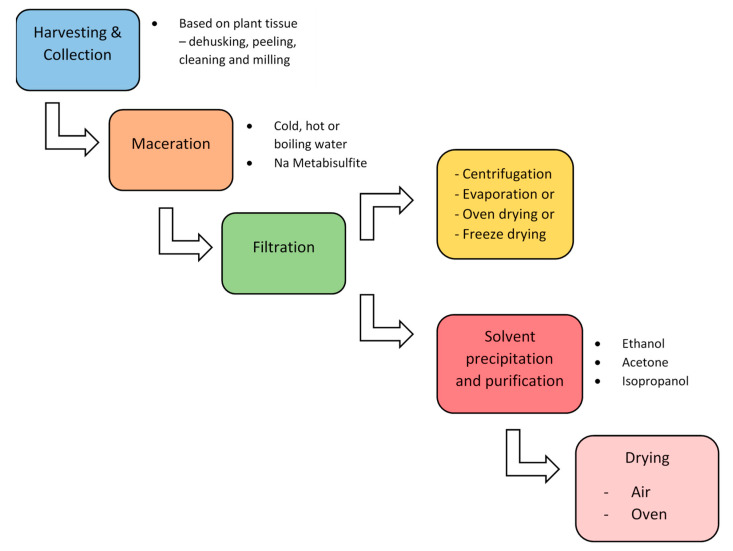
Schematic diagram of the general process of extraction of plant polymers optimized according to reference [32].

Polymers derived from plants are obtained by soaking parts of the plant, such as the leaves, roots, seeds, or fruits, in water at ambient or high temperatures. The gum or mucilage is then separated from the plant part through filtration methods like a muslin bag. The mucilage is separated from water by adding alcohol, typically absolute ethanol, as it provides a higher yield than ethanol: water mixture precipitates faster. Ethanol is also preferred because it is eco-friendly and FDA-approved [32,74]. Other solvents like acetone and methanol are also used for mucilage precipitation. The mucilage is then dried through air-drying or oven-drying. However, instead of precipitation with alcohol, Ahuja and co-workers decided to dry the mucilage. They macerated *Mimosa pudica* seeds in water for ten hours and then dried the obtained mucilage, including the seeds, in the oven for approximately 4–5 h at 50 °C. Later, the dried mucilage was separated from the seeds by passing it through № 18 mesh, and the seed husks were removed by winnowing. Some people prefer to freeze-dry the mucilage instead of heating it (Figure 10) [32,74].

## 5. Synthetic Polymers for Biomedical Use

### 5.1. Antiviral

Polymers are widely used in surface coating applications due to their long chain length or high molecular weight. They can form many physical bonds with the surface, resulting in irreversible coating. This makes them an attractive material for antiviral purposes because they can bond irreversibly with viral glycoproteins, covering and concealing the viral surface and preventing interaction with host cells [33]. Synthetic polymers offer an advantage over natural ones since they can be engineered to maximize antiviral activity against specific viruses. The chemical composition, functional group type and extent of functionalization, molecular weight, charge density, distribution, degradation, and stability can all be tailored to enhance antiviral properties. Dendrimers and sialyl-based polymers are synthetic polymers extensively researched as antiviral agents against infections [33].

### 5.2. Antibacterial

**Figure 11:**
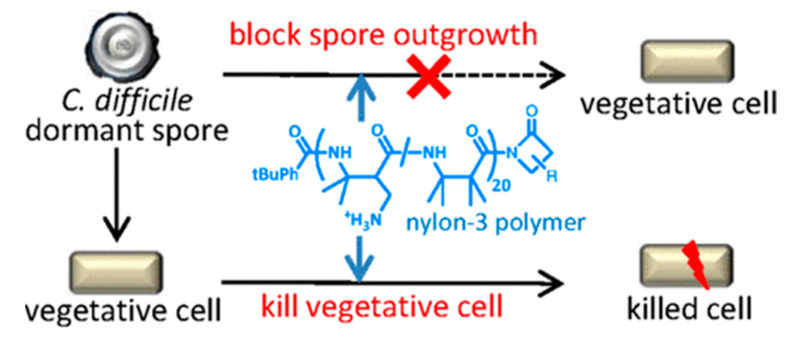
Schematic representation of nylon-3 polymer’s activity against *C. difficile* [75].

In a study by Lui et al., nylon-3 polymers were evaluated for inhibitory activity toward *C. difficile* [75]. Some of these polymers were previously shown to be active against other pathogenic bacteria, including methicillin-resistant *Staphylococcus aureus* (MRSA), vancomycin-resistant *Enterococcus faecium* (VREF), *Salmonella enterica* LT2, *Bacillus cereus* ATCC14579, *Pseudomonas aeruginosa* PA1066, and uropathogenic *E. coli* CFT073. These polymers inhibit the growth of the pathogen’s vegetative form and prevent the outgrowth of the spore form of *C. difficile*. Preventing the development of vegetative cells is critical to controlling infection and stopping the production of toxins that lead to human disease. Preventing the outgrowth of spores has the added advantage of avoiding vegetative growth entirely (Figure 11) [75].

In a study by Zhang et al., a novel hydrogel dressing with polyasparthydrazide (PAHy) nanofibers and silver nanoparticles (AgNPs) demonstrated excellent antibacterial activity against *E. coli* and *S. aureus*. The hydrogel mat also showed a great ability to promote wound healing in animal studies by promoting re-epithelialization and collagen deposition [76].

### 5.3. Antifungal

Encapsulating drugs into functionalized polymeric nanoparticles (NPs) is a new alternative to reach the specific therapeutic target with lower doses. However, when NPs come into contact with physiological media, proteins adsorb on their surfaces, forming a protein corona (PC) biomolecular layer and acquiring a distinct biological identity that alters cell interactions. Mejía et al. tested Itraconazole (ITZ), an antifungal agent, encapsulated into PEGylated and functionalized NPs with high specificity for macrophages [77]. Minimum inhibitory concentration (MIC) and colony-forming unit assays demonstrated that encapsulating ITZ into poly (ethylene glycol) (PEG) NPs improves the antifungal effect compared to NPs lacking PEG. The improvement can be due to the synergistic effect of the encapsulated ITZ and NP composition and the reduction of PC formation in PEG NPs [77].

### 5.4. Antitumor

Our experimental contribution is explained in one of our papers, which reveals that PEO-b-PnBA-b-PAA triblock terpolymers, comprising a relatively longer PEO and much shorter PAA outer blocks, are promising precursors for the preparation of multifunctional nano-carriers foreseen for multi-drug therapy application [78]. The formation of a multilayer micellar structure, consisting of a hydrophobic PnBA core, a PEO/PAA middle layer, and a hydrated PEO outer layer, was exploited to load two anti-cancer agents within the carrier, preserving the colloidal stability of the system. The co-existence of AgNPs and curcumin significantly increased the agents’ cytotoxic activity compared to individual drug/AgNP-loaded carriers. The combined administration of two active agents allows for minimizing the amount of each drug and eventually suppressing the drug resistance through different action mechanisms [78].

Synthetic biomaterials have also been used to form multicellular tumor spheroid models (MCTSs). MCTSs are often created as 3D in vitro models that can mimic the microenvironment of tissues [34]. MCTSs have gained increasing interest in nano-biotechnology as they can provide easily accessible information on nanoparticle performance without animal models. Synthetic polymers offer several advantages over natural ECMs, including more tunable stiffness, cell ligand density, and other biochemical properties. However, these synthetic materials are biologically inactive and thus must be functionalized with cell adhesion peptide domains to encourage cell adhesion and crosslinked to form biodegradable bonds for cell remodeling of the ECM [34].

Lastly, Wang et al. designed a nanomotor with integrated fluorescence and therapeutic potential based on biodegradable polymersomes equipped with aggregation-induced emission (AIE) agents [79]. AIE segments provide the polymersomes with autofluorescence, facilitating the visualization of cell uptake. These polymersomes display fluorescence upon laser irradiation and produce reactive oxygen species (ROS). As ROS are also used for cancer cell treatment, polymersomes act as delivery vehicles and therapeutic agents [79].

### 5.5. Myocardial Tissue Engineering

Synthetic polymeric scaffolds are excellent candidates for cardiac patch tissue engineering because they are easily tailored and fabricated to fit the particular needs of native tissues [37]. Polymers have a wide range of mechanical properties and good biocompatibility, and their degradation rate can be easily manipulated. Additionally, synthetic polymers are known for their durability, porosity, and microstructure and can be tailored to meet the specifications of natural cardiac tissues. Though polymers as biomaterials can lead to reduced cell adhesion and scaffold integration, specific modifications, such as adding stimuli and growth factors, maintain their popularity as candidates for tissue scaffolds. Polymer chain variabilities lie in the chemical structures, molecular weights, molecular weight distribution, and functional groups that can be attached to the polymer [37]. Cardiac scaffolds require a certain degree of elasticity and mechanical strength to withstand the dynamic nature of the heart. Many cardiac scaffolds developed incorporate a combination of polymers to achieve these properties. Blending polymers in scaffolds combines the mechanical properties of different polymers to form a scaffold with many desirable characteristics. Several biodegradable polymers, such as poly(ε-caprolactone) (PCL), poly (glycerol sebacate) (PGS), poly (lactic-co-glycolic acid) (PLGA), biodegradable polyurethane (PU), and poly(l-lactide) (PLLA), are common polymers of interest for cardiac patch application research [80].

### 5.6. Insulin Drug Carriers

**Figure 12:**
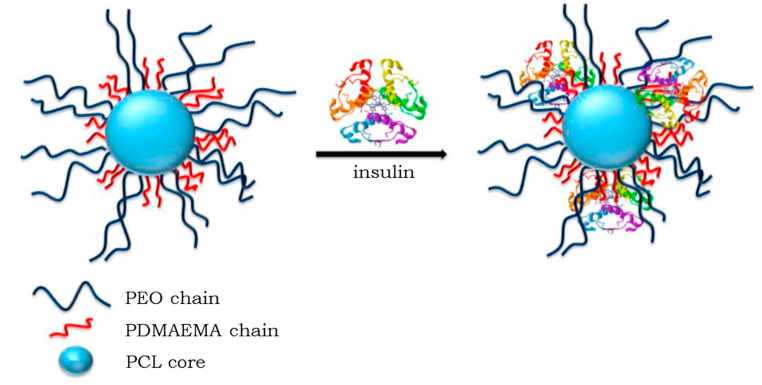
Schematic representation of micelle and its complexation with insulin [81].

In another study conducted by us, experimental results and research show that block copolymer micelles (BCMs) are among the most studied nanocarriers of various low-molar-mass therapeutic substances and biomacromolecules intended to treat numerous diseases [81]. The main advantages of BCMs compared to other polymeric carriers are their small size, high colloid stability in vitro and in vivo, low toxicity, and potential to effectively dissolve and deliver hydrophobic bioactive substances to target organs/tissue in a controlled manner (Figure 12).

Several peptides and proteins have emerged as promising therapeutic agents for treating cancer, diabetes, anemia, hemophilia, etc. A significant obstacle to the controlled delivery of these agents to target sites often arises from the large size of molecules, sensitivity to denaturation and degradation, short half-life, and poor bioavailability [81]. One of the promising strategies to solve these problems is to use polymeric nanocarriers since they have several advantages over conventional delivery systems (e.g., tablets, capsules, beads, microparticles, and microemulsions). Incorporation into polymer nanocarriers protected insulin molecules from degradation in the case of proteins like insulin. It facilitated their uptake via transcellular and paracellular pathways, increasing therapeutic efficacy. In addition to the variety of insulin-loaded particles developed in the last two decades via the complexation of insulin with positively charged polymers, polymeric micelles have attracted particular attention as highly desirable carriers of insulin because of their characteristics (nanoscale size, protective shell, and fictional groups), which favor more extended circulation stability, more straightforward renal clearance, and controlled release of insulin [81].

### 5.7. Biofilm

Personal experimental results in this field showing the effectiveness of cationic polymer micelles (CPMs) based on newly synthesized di- and triblock copolymers were tested to destroy pre-formed biofilms of Gram-negative and Gram-positive bacterial strains [82]. Block copolymers based on quaternized poly (2-vinylpyridine) (PQVP) and poly (2-(dimethylamino)ethyl methacrylate) (PDMAEMA) cationic moieties were used. Biofilms of *Escherichia coli* 420, *Pseudomonas aeruginosa* PAO1, *Staphylococcus aureus* 29213, and *Bacillus subtilis* 168 were cultivated for 24 h, then the pre-formed biofilms were treated with CPMs for 2, 4, or 6 h. Treatment with CPMs resulted in a reduction in the biomass of the pre-formed biofilms. The promising effects of the tested CPMs have been confirmed on the model of four single-species biofilms, two Gram-negative and two Gram-positive strains with good biofilm-forming potential. This implies the applicability of the CPMs to medically necessary biofilms [82].

The study shows that polymeric cationic micelles can remove bacterial biofilms from contaminated surfaces in hospitals and the food processing industry, decontaminate medical devices, and treat surface-exposed biofilm-related skin lesions. Of the tested micelles, MKPa4 and MKPa12 are applicable against Gram-negative and Gram-positive bacteria, while PSPQ2VP 35 and PSPQ2VP 115 are effective against Gram-positives only. The micelles are also expected to have the potential for drug delivery within biofilms [82].

### 5.8. Gene Delivery

A novel gene delivery system, described in detail in one of our papers, encapsulates a polyplex between pDNA and cationic micelles with a biodegradable crosslinked shell. The outer shell can provide additional functionality to the system and protect DNA from degradation. The encapsulated polyplex exhibits a similar transfection efficiency to the naked polyplex [83].

In a study by Kaygisiz et al., non-pathogenic viral particles are described as promising prospective vectors for delivering genetic material into cells in the context of gene therapy and vaccines [84]. Lentiviruses and γ-retroviruses are the vectors of choice in fundamental research as well as most clinical trials that are currently underway. However, low concentrations of viral vectors must be used to avoid side effects such as cytotoxicity and immunogenic reactions. Therefore, efficient virion-cell attachment is essential for gene transduction, which remains a significant challenge in retroviral gene delivery. In ex vivo clinical applications, preventing exposure to possibly harmful substances to the patient is paramount. Therefore, any aggregated adjuvants should be removed or degraded after successful transduction. Transduction-enhancing additives have been reported to overcome these challenges, including synthetic polymers, lipids, peptides, and others [84].

### 5.9. Bioink in 3D Printing

Three-dimensional (3D) printing technologies enable manufacturing processes that automatically produce complex structures directly from computer-aided design (CAD) models with high resolution and sophistication [38]. These technologies are based on a layered manufacturing paradigm that builds solid objects by incremental material deposition and fusion of thin cross-sectional layers. By breaking down complex 3D shapes into simpler two-dimensional (2D) layers, assembling very complex structures can be dramatically simplified under the instructions of CAD models. Three-dimensional printing technologies are considered the most convenient and reliable technique for manufacturing bioartificial organs with multiple types of cells and other biomaterials [38].

**Figure 13:**
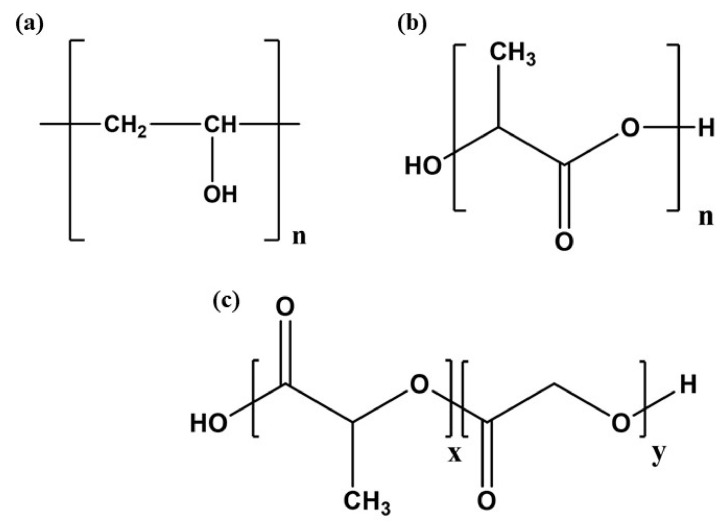
Chemical structure of synthetic polymers: (**a**) polyvinyl alcohol (PVA), (**b**) polylactic acid (PLA), and (**c**) poly(lactic-co-glycolic) acid (PLGA) [39].

As the main components of ‘bioinks,’ polymers have played a critical role in organ 3D printing during layered 3D construction processes. Most ‘bioinks’ are cell-laden polymeric hydrogels, which are usually formed through physical (reversible), chemical (reversible or irreversible), or biochemical (irreversible) crosslinking of homopolymer or copolymer solutions. Cell behaviors within polymeric hydrogels can be controlled by changing the physical and chemical properties of the employed polymers. Polymeric hydrogels used for organ 3D printing include natural and synthetic polymers and their combinations. Natural polymeric chains are entirely bioactive groups that can provide a benign and stable environment for cells, mainly stem cells, to grow, migrate, increase, and differentiate inside. Synthetic polymeric networks are comprised of repeatable inert units. They are usually superior to natural polymers regarding mechanical properties and immunogenic responses. The most commonly printed synthetic polymers include poly (lactic acid) (PLA), poly (glycolic acid) (PGA), polylactic-co-glycolic acid (PLGA), polyurethane (PU), and polycaprolactone (PCL) (Figure 13) [38].

### 5.10. Textiles in Medicine

Textiles in medicine, such as non-implantable textiles, are mainly used for external applications outside the human body for surface wound treatments of different parts of the human body [40]. They protect against infection, absorb blood and exudates, and promote healing. They include wound dressings, bandages, plasters, absorbent pads, gauze, wadding, pressure garments, orthopedic belts, heating pads, etc. Common types of medical textiles for wound healing are made from different kinds of fibers, such as cotton and silk, and natural and synthetic polymers, like polyester, polyamide, PP, polyurethane, polytetrafluoroethylene, alginates, proteins, poly-glycolic acid, regenerated cellulose, chitin, and chitosan [40].

Compared with natural polymers, synthetic polymers have good mechanical properties and thermal stability and can be more easily processed into different forms with controlled batch-to-batch consistency during production. Depending on their application, synthetic polymers have high tunability properties, such as strength, flexibility, degradation rate, resistivity, and chemical inertness. They are cheaper for producing fibers and yarns. The degree of control over synthetic polymers offers excellent versatility for many different applications. On the other hand, biopolymers typically have better biofunctions and biodegradation properties [85]. In some applications, natural–synthetic blends are of interest, as they can combine the advantages of each polymer type while avoiding their disadvantages [86,87].

In a recent review by Li et al. [35], surgical suture materials from both synthetic and natural polymers are discussed, along with their advantages and disadvantages. The review covers various fiber fabrication strategies and advanced designs for the development of surgical sutures with multiple functionalization, such as surface-coating and drug-loading technologies.

## 6. Synthetic Polymers for Environmental Use

### 6.1. Agriculture

Synthetic polymers play an essential role in agriculture, such as structural materials for creating a climate beneficial to plant growth, e.g., mulches, shelters, or greenhouses, fumigation, and irrigation in transporting and controlling water distribution. However, the principle requirement for polymers used in these applications concerns their physical properties, such as stability, permeability, transmission, or weatherability, as inert materials rather than active molecules [88]. In recent years, there has been significant interest in the science and technology of reactive functionalized polymers, considered one of the most fascinating areas of polymer chemistry. These polymers have gained popularity due to their ability to produce improved materials. This is because of the active functional groups and the characteristic properties of polymeric molecules. Reactive functionalized polymers have found broad applications in various fields, such as solid-phase synthesis, biologically active systems, and different technological uses [88].

### 6.2. Food Packaging

Traditional polymers used in food packaging applications include polyethylene (PE), poly(ethylene terephthalate) (PET), or polystyrene (PS), among others [89]. These polymers protect against chemical, biological, and physical damage and prevent the loss of flavor, aroma, and antioxidants. They also ensure an adequate balance of gases and humidity inside the packaged food, increasing its shelf life and facilitating its handling [41].

### 6.3. Hazardous Waste Management

Cesium-137 (137Cs), a primary component of intermediate-level radioactive nuclear waste, poses a significant hazard due to its intense radioactivity and long half-life. In particular, Cs^+^ in its ionic form can penetrate organisms and dissolve into natural water bodies, causing irreversible damage and persistent radioactive pollution. Three-dimensional-printed geopolymer lattices (3DGPLs) have recently been considered promising for hazardous waste management due to their low cost, porous nature, and excellent environmental stability [90]. Geopolymers (GP) are a class of inorganic polymers composed of a SiO_4_/AlO_4_ tetrahedral framework and countercations. In a study by Siqi Ma et al., the Cs^+^ adsorption and immobilization properties of 3DGPLs in the presence of competing ions and gamma-ray radiation were observed. The results reveal the inhibitory intensity of Cs^+^’s adsorption capacity by five common metal cations in seawater, ranked in the following order: Na^+^ > K^+^ > Ca^2+^ > Mg^2+^ > Sr^2+^ [90].

Water and wastewater treatment have also been possible using molecularly imprinted polymers (MIPs), custom-made materials with specific recognition sites for a target molecule. Their specificity and the variety of materials and physical shapes in which they can be fabricated make them ideal components for sensing platforms. Before polymerization, the target analyte, or template, is combined with a functional monomer to form a precursor structure by covalent, semi-covalent, or non-covalent bonding [42]. Then, they are polymerized in the presence of a crosslinker and an initiator in a porogenic solvent. Afterward, the template is eluted by extraction with a proper solvent or chemical cleavage to create empty recognition cavities in the polymer matrix, whose morphology and functionality complement the template molecule. In addition to water and wastewater treatment, MIPs have also been fabricated for solid-phase extraction, chromatographic separation, catalysis, drug delivery, the study of the structure and function of proteins, environmental and biomedical sensing, and membrane-based separations [42].

### 6.4. Phenol Degradation

Phenol and its numerous derivatives are widely exploited in the chemical industry, agriculture, and wood processing. They are highly toxic, mutagenic, and teratogenic, and some of them have been reported as potential carcinogens. Phenol is a primary raw material in the chemical industry and a byproduct of benzene processing. The chemical, wood, textile, and oil-processing industries release it into the environment. The world’s production reaches seven million tons per year [91]. Many bacteria belonging to the genera *Pseudomonas*, *Acinetobacter*, *Alcaligenes*, *Bacillus*, *Sphyngomonas*, and *Geobacter*, as well as some fungal species that belong to *Aspergillus*, *Trichosporon*, or *Candida*, are known to degrade phenol. Few studies have been reported on phenol degradation by immobilized bacteria. These include reports on immobilizing microbes on polymers such as polyacrylamide, polyurethane, polyamide, polyacrylonitrile, or polyvinyl alcohol. Recent investigations demonstrated that synthetic polymers are the most appropriate materials for microbial immobilization, especially for biotechnological applications [91]. Our study showed that two environmental strains, *Pseudomonas rhodesiae*, KCM R5, and *Bacillus subtilis*, RG5, were successfully entrapped in PEO cryogels. The strains were isolated from heavy metal and pesticide-polluted soils and showed phenol removal at concentrations of 1000 mg L^−1^ for a period of 30 days for *Pseudomonas rhodesiae*, KCM R5/PEO cryogel, and 600 mg L^−1^ for a period of 17 days for *Bacillus subtilis*, RG5/PEO cryogel [91].

Immobilized bacteria possess some advantages over free-swimming or planktonic cells. They harbor higher genetic capability due to the increased plasmid transfer in the microbial biofilms produced during cell immobilization. Also, cell immobilization is biotechnologically easier because of facilitated process control.

Among synthetic polymers, poly (ethylene oxide) (PEO) hydrogels are excellent candidates for bacterial immobilization because they are nontoxic, biocompatible materials and meet all strength, absorbance, flexibility, and adhesiveness requirements [91]. PEO hydrogels were first obtained in situ by γ-irradiation of dilute aqueous solutions and later via methods based on chemical cross-linking. Hydrogels of high molecular weight (MW) PEO are also easy to synthesize in situ by ultraviolet (UV) cross-linking of PEO in aqueous solution. Moreover, when UV cross-linking is carried out in a frozen aqueous system, super-macroporous hydrogels (cryogels) with a very high yield of gel fraction (GF) and high cross-linking density can be obtained [91].

### 6.5. Soil Stabilizers

Soil is a common element in geotechnical works, exhibiting significant variation and stratification among regions, often necessitating stabilization and, consequently, improvement of the underlying substrate [92]. Many geotechnical projects aim at soil stabilization, wherein the success of soil enhancement procedures is evaluated, striving to promote soil stability, strength, resistance to erosion, and economic viability. This holds for stabilization, improvement, or reinforcement of diverse geotechnical structures.

Synthetic polymers, being artificial, can be manipulated to acquire desired characteristics. Consequently, many synthetic polymers are being developed to be less harmful to the environment (from production to use), more durable, and economically viable for soil stabilization [93].

The prioritization of environmentally friendly materials to enhance soil quality has become widespread, driven by increasing environmental awareness. With growing concern about environmental impact, various investigations are being conducted into new “eco-friendly” materials. Using polymers as modifiers of soil structures appears promising, improving mixtures’ microstructure and enhancing composites’ durability [94,95].

## 7. Methods of Synthesis and Physicochemical Parameters of Synthetic Polymers

Chemically synthesized polymers, including polylactide (PLA), polyurethane (PU), poly (lactic-co-glycolic acid) (PLGA), poly (methyl methacrylate), silicone rubber, polyester, polyvinyl alcohol, polyvinyl pyrrolidone, and so on, are utilized as materials in the biomedical field and have been developed via chemical techniques.

PLA and its copolymers are biodegradable and biocompatible and can be acquired from a broad range of raw material sources. PLA is non-toxic, renewable, and biodegradable and possesses good thermal formability, mechanical strength, and elastic modulus [96]. It is utilized in cartilage regeneration, repairing cartilage, bone tissue engineering, and controlled drug release formulations that function as carriers. A continuous drug-releasing system based on PLA provides gradual drug release through its moderate degradation rate in vivo. The PU material comprises excellent fatigue resistance, good compatibility (blood, biological, and tissue compatibility), high elasticity, high strength, and wear resistance compared to other polymeric materials. Therefore, in the biomedical field, materials such as PU are widely utilized for the fabrication of polymeric capsules of drugs, the development of artificial organs, and catheter interventions. The essential properties of PU are low toxicity, excellent clotting, nonallergic, noncarcinogenic, and nonteratogenic. PLGA has good biodegradability and biocompatibility and is used broadly in preparing NPs, microspheres, pellets, microcapsules, films, and implants [43]. As another kind of controlled drug release material, copolymers of PLGA have been broadly utilized in the controlled release of antibiotic drugs, chemotherapeutic agents, peptides, proteins, polysaccharides, and different drugs [97]. Table 3 summarizes some synthetic polymers, their methods of synthesis, and their application.

**Figure 14:**
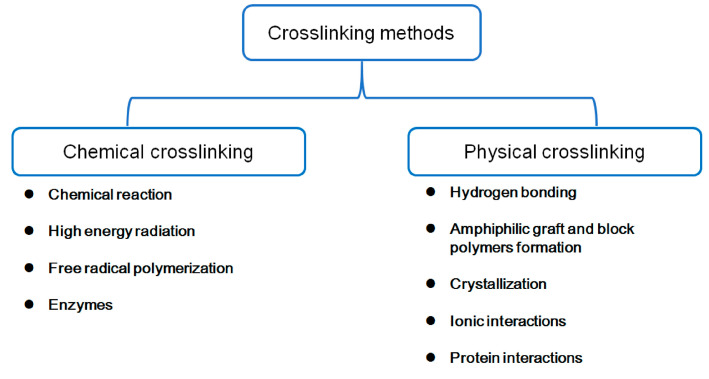
Chemical and physical methods of crosslinking [50].

Hydrogel fabrication is needed to design drug-delivery systems, which are carried out using natural or synthetic polymers and applying various techniques to accomplish the objective of established crosslinking [44]. Crosslinking methods used in hydrogel fabrication are divided into physical and chemical and are presented in Figure 14. Physical crosslinking refers to noncovalent interaction and/or entanglement between the polymer chains, while chemical crosslinking occurs through covalent bond formation [44].

## 8. Advantages and Limitations: Future Perspectives of Natural and Synthetic Polymers

Over the past few decades, natural polymers have resurged as primary bioactive substances used in the application of medical materials. Biofunctional molecules that ensure bioactivity, biomimetic nature, and natural restructuring are typically found in such polymers [45]. Bioactivity, biocompatibility, 3D geometry, antigenicity, non-toxic byproducts of biodegradation, and intrinsic structural resemblance are the most essential properties of natural polymers. Conversely, their key disadvantages, microbial contamination (i.e., endotoxins), decreased tunability, immunogenic reaction, uncontrollable rate of degradation, and poor mechanical strength, restrict their application for complex tissue regeneration. Natural polymers are essential to tissue engineering, especially in manufacturing scaffolds for therapeutic agent delivery. Novel natural polymeric materials are aimed at enhancing different therapies due to their inherent bioactivity, biocompatibility, and bioresorbability [36].

Synthetic polymers are advantageous in a few characteristics, such as tunable properties, endless forms, and established structures over natural polymers. The support offered by synthetic biomaterials can enable the restoration of damaged or diseased tissue structure and function. Polymerization, interlinkage, and functionality (changed by block structures, combining them, and copolymerization) of their molecular weight, molecular structure, and physical and chemical features make them easily synthesized compared to naturally occurring polymers [98]. The disadvantages of synthetic biomaterials are that they lack cell adhesion sites and require chemical modifications to enhance cell adhesion. Many commercially available synthetic polymers exhibit similar physicochemical and mechanical characteristics to biological tissues. Synthetic polymers are produced from hydrocarbon building blocks in the laboratory setting. Although the intrinsic cell interaction moieties of the biopolymers may be lacking, their capacity to be controlled explicitly in structure and reproducibility make them useful along with natural polymers in biomaterial composites for tissue engineering applications [36]. Table 4 summarizes some representative natural and synthetic polymers and their advantages, disadvantages, and perspectives.

Various biopolymers compete successfully in the global market due to their unique characteristic properties, which have a considerable demand in biomedical applications. Furthermore, it is no surprise that improving human health and lifespan contributes to one of the fastest-growing markets for tissue engineering and regenerative medicine products. To help with this, the industry has been developing new biomaterial-based products, including synthetic and naturally derived materials [36]. Also, norbornene derivatives (NBEs) have recently been used as typical monomers for living ring-opening metathesis polymerization and yield polymers with low dispersity and diverse functionalities. However, the all-carbon backbone of poly-NBEs is non-degradable. Researchers have reported a new method for producing degradable polymers by copolymerizing 2,3-dihydrofuran with NBEs. This reactivity reduces NBE homoaddition, which leads to the uniform incorporation of acid-degradable enol ether linkages throughout the copolymers. As a result, complete polymer degradation can occur while retaining the favorable characteristics of living ring-opening metathesis polymerization. These polymers can be broken down into small molecules or oligomeric species under mildly acidic conditions. This technique is easily adaptable to the conventional ring-opening metathesis polymerization of widely used NBEs, making it possible to create easily degradable polymers with adjustable properties for various applications and environmental sustainability [99].

## 9. Conclusions

This review provides a comprehensive and extensive overview of natural and synthetic polymers used for biomedical and environmental applications. In contrast to other reviews on the topic, this paper also describes methods for their isolation and synthesis. In addition to the extensive and detailed literature overview, this work is valuable as it also includes the original results of the authors, who published six papers for both biomedical and environmental contributions. The review describes the vast use of natural polymers and their application as carriers for drug delivery, tissue engineering, stem cell morphogenesis, wound healing, regenerative medicine, food packaging, bioplastics, etc. They possess numerous essential properties, such as bioactivity, biocompatibility, 3D geometry, antigenicity, non-toxic byproducts of bio-degradation, and intrinsic structural resemblance, and thus have promising prospects. On the other hand, some identified problems and limitations of natural polymers include the possibility of microbial contamination, decreased tunability, immunogenic reaction, uncontrollable rate of degradation, and poor mechanical strength. Synthetic polymers are also widely applied in controlled drug-release systems, tissue engineering, nano-carriers, dispersion of bacterial biofilms, gene-delivery systems, bio-ink in 3D printing, textiles in medicine, agriculture, heavy metals removal, food packaging, etc. Some advantages are their tunable properties and endless forms, which can restore damaged or diseased tissue structure and function. Polymerization, interlinkage, and functionality of their molecular weight, molecular structure, and physical and chemical features give synthetic polymers promising prospects. Some of their identified problems are that they lack cell adhesion sites and require chemical modifications to enhance cell adhesion. Natural and synthetic polymers will continue to be used as they are irreplaceable resources with diverse benefits. Some of our future work with polymers for biomedical applications will include synthesis and research on multifunctional nanocarriers, such as polymer micelles, nanogels, polymerzomes with antimicrobial peptides, enzymes, DNA, and other biomolecules entrapped in them.

## Figures and Tables

**Table 1:** Comparison between the natural and synthetic polymers’ parameters. Optimized according to reference [9].

Natural Polymers	Synthetic Polymers
In use for millions of years	First produced 125 years ago
Similar or nonidentical repeating units	Identical repeating unit
Properties are naturally controlled	Properties are engineered
Usually biodegradable	Some are biodegradable
The backbone structure is carbon, oxygen, and nitrogen	The backbone is mostly carbon
Environmentally friendly	Some are friendly, and some are toxic to the environment
Limited recyclability	Most of them are capable of being recycled multiple times

**Table 2:** Characteristics of some natural bio-based monomers used in biomedical applications optimized according to reference [18].

Bio-Based Monomers	Source	Characteristics
Itaconic acid	*Aspergillus itaconicus*	Antimicrobial activity, non-toxic, biocompatible, biodegradable, chemical reactivity, surfactant forming ability, hydrophilic activity, wound-healing activity, coating forming ability, water uptake ability, drug carrier ability, and hydrogel-forming ability
Succinic acid	*Actinobacillus succinogenes*, *Anaerobiospirillum*, and *Mannheimia succiniciproducens*	Biocompatible, biodegradable, non-toxic, chemical reactivity, food additives ability, food flavoring ability, surfactant/detergent extender/foaming ability, drug carrier ability, pH control ability, antimicrobial activity, and corrosion prevention ability
Citric acid	Citrus fruits and *Aspergillus niger*	Biocompatible, biodegradable, non-toxic, excellent chelating property, anti-odor property, chemical reactivity, pH control ability, food additives ability, food flavoring/preservative ability, and drug carrier ability
Glutamic acid	*Bacillus subtilis* and *Bacillus licheniformis*	Biodegradable, biocompatible, non-toxic, excellent chelating property, heavy metal removal ability, cosmetic property, drug carrier ability, hydrophilic activity, anionic property, thickener property, aging inhibitor ability, and use as an additive

**Table 3:** Synthetic polymers, including their methods of synthesis and application.

Synthetic Polymer	Method of Synthesis	Application
Polylactide	Ring-opening polymerization of lactide; Polycondensation of lactic acid	Cartilage regeneration; repairing cartilage; bone tissue engineering; controlled drug release formulations that function as carriers; continuous drug-releasing systems
Polyurethane	Polyaddition reaction between diols and diisocyanates	Fabrication of polymeric capsules of drugs; myocardial tissue engineering; textiles in medicine; bioink in 3D-printing; phenol degradation
Poly(lactic-co-glycolic acid)	Ring-opening copolymerization; Polycondensation of lactic acid and glycolic acid	Myocardial tissue engineering; bioink in 3D-printing; drug-releasing systems
Poly (methyl methacrylate)	Free-radical polymerization of methyl methacrylate	Prosthetic dental applications; soft contact lenses; bone tissue regeneration; drug delivery and controlled drug-delivery systems
Silicone rubber	Crosslinking of poly(dimethyl siloxanes)	Medical implants; electrical insulation; waterproof coating
Poly(vinyl alcohol)	Hydrolysis of polyvinyl acetate	Used in drug production; textiles
Poly(vinyl pyrrolidone)	Free radical polymerization of vinylpyrrolidone	Cartilage regeneration

**Table 4:** Naturally occurring and synthetic biopolymers and their advantages, disadvantages, and perspectives optimized according to reference [36].

Polymer	Advantages	Disadvantages	Perspectives
Collagen *	Good for cell adhesion, proliferation, differentiation, ECM secretion; excellent biocompatibility; biodegradability; low toxicity; rough surface morphology; low immunogenicity; weak antigenicity	Low mechanical strength; difficult disinfection; deformation of collagen-based scaffolds restricts their use in load-bearing tissues; poor stability in aqueous environments; potential for antigenicity through telopeptides	Drug delivery; wound healing; tissue engineering
Gelatin *	Infiltration, adhesion, spreading, and proliferation of cells on resulting scaffolds; good stability at high temperatures in a broad pH range; biodegradability; osteoconductivity; non-immunogenic; low antigenicity	Low bioactivity in higher-order gelatin structures in scaffolds; low stability in physiological conditions	Antibacterial; wound healing; tissue engineering
Starch *	Biocompatible; thermoplastic; non-cytotoxic; guides various developmental stages of cells; hydrophilicity; substrate for cell adhesion; good biodegradation period	Very high water uptake; low mechanical strength; unstable for long-term application; chemical modifications can lead to toxic byproducts and reduce the degradation rate	Bioplastics
Chitin/chitosan *	Accelerates tissue repair; prevents the formation of scar tissue; promotes cell adhesion; non-toxic and non-allergenic; bioactivity; anti-inflammatory; osteoconductivity; hemostatic potential; scaffolds could be used for a more extended period; chitosan-based scaffolds can immobilize growth factors	Poor mechanical strength and stability; high viscosity and low solubility at neutral pH; rapid in vivo degradation rate	Textiles in medicine; nano carriers used for nano fertilizers and micronutrients; antibacterial; drug delivery; wound healing; tissue engineering
Cellulose *	Stable for tissue engineering applications; good mechanical strength; hydrophilicity; biocompatibility; cytocompatibility; bioactivity	In the human organism, it behaves as a nondegradable or very slowly degradable material	Heart valve tissue engineering; nano carriers used for nano fertilizers and micronutrients; bioplastics; textiles in medicine
Polylactic acid (PLA) **	Biocompatible; cytocompatibility; thermal stability; excellent mechanical strength; good degradation rate; non-toxic degradation products	PLA-based materials lack ideal surface chemistry for cell adhesion and proliferation; brittleness; poor thermal stability; hydrophobicity	Bioplastics; bioink in 3D-printing; drug-releasing systems
Polylactic-co-glycolic acid (PLGA) **	Excellent cell adhesion and proliferation; good mechanical properties; wide range of degradation rates	Poor osteoconductivity; may develop biocompatibility problems	Myocardial tissue engineering; bioink in 3D-printing; drug-releasing systems
Polyglycolic acid (PGA) **	Biocompatible; high tensile modulus; high melting point; undergoes bulk degradation; hydrophilicity	High sensitivity to hydrolysis; challenging to obtain porous PGA scaffolds without toxic solvents	Bioink in 3D-printing
Poly(ethylene glycol) (PEG) **	Bioadhesive; mucoadhesive; hinders protein adsorption; hydrophilic; can be modified to different moieties to pass different requirements of a skin substitute like cell adhesion, short-term degradation, and minimum inflammation; non-immunogenic	Lacks cell-interactive character due to its bio-inert nature; nonreactive, creates insoluble networks	Antifungal
Polyvinyl alcohol (PVA) **	Biocompatible, nontoxic, and noncarcinogenic, it displays a reduced protein-binding tendency, relatively higher elasticity, and water content. It is a highly hydrated water-soluble synthetic polymer with relatively similar tensile strength to human articular cartilages and good lubrication	Lack of cell-adhesive property; less ingrowth of bone cells	Used in drug production; textiles

*—Natural polymer; **—Synthetic polymer.

## Data Availability

Data are contained within the article.
